# Eupatilin Exerts Antinociceptive and Chondroprotective Properties in a Rat Model of Osteoarthritis by Downregulating Oxidative Damage and Catabolic Activity in Chondrocytes

**DOI:** 10.1371/journal.pone.0130882

**Published:** 2015-06-17

**Authors:** Jeong-Hee Jeong, Su-Jin Moon, Joo-Yeon Jhun, Eun-Ji Yang, Mi-La Cho, Jun-Ki Min

**Affiliations:** 1 The Rheumatism Research Center, Catholic Research Institute of Medical Science, The Catholic University of Korea, Seoul, South Korea; 2 Division of Rheumatology, Department of Internal Medicine, College of Medicine, The Catholic University of Korea, Seoul, South Korea; Indian Institute of Integrative Medicine, INDIA

## Abstract

Increases in oxidative stress are thought to be associated with the development of osteoarthritis (OA). Eupatilin, one of the major compounds present in *artemisia species*, was shown to have both anti-oxidative and anti-inflammatory properties. Here, we investigated the *in vivo* effects of eupatilin on pain severity and cartilage degradation in an experimental rat model of OA, along with the mechanisms of action underlying these effects. Experimental OA was induced via an intra-articular injection of monosodium iodoacetate (MIA), with oral administration of eupatilin initiated on the day of MIA injection. Pain was assessed by measuring the paw withdrawal latency and threshold. Cartilage destruction was analyzed macroscopically and histomorphologically. The effects of eupatilin on mRNA expression were investigated in interleukin-1β (IL-1β)-stimulated human OA chondrocytes. Eupatilin treatment exhibited clear antinociceptive effects, along with an attenuation of cartilage degradation in OA rats. Additionally, the number of osteoclasts present in the subchondral bone region was significantly decreased following eupatilin treatment. Eupatilin reduced the expression of interleukin-1β (IL-1β), interleukin-6 (IL-6), nitrotyrosine and inducible nitric oxide synthase (iNOS) in cartilage. mRNA levels of matrix metalloproteinase-3 (MMP-3), MMP13, and a disintegrin and metalloproteinase with thrombospondin motifs-5 (ADAMTS-5) were reduced in IL-1β-stimulated human OA chondrocytes, while tissue inhibitor of metalloproteinases-1 (TIMP-1) was induced. Phosphorylated protein levels of the c-jun N-terminal kinase (JNK) was reduced by eupatilin. Taken together, these results suggest that eupatilin suppresses oxidative damage and reciprocally enhances extracellular matrix production in articular chondrocytes, making eupatilin a promising therapeutic option for the treatment of OA.

## Introduction

Osteoarthritis (OA) is the most common form of arthritis characterized by a progressive loss of articular cartilage, osteophyte formation, and changes within subchondral bones, resulting in debilitating chronic pain in affected individuals. While OA has long been defined as a degenerative disease characterized by increased pressure on a particular joint, the current understanding of OA has shifted from cartilage “wear and tear” to an inflammatory joint disease [1]. Proinflammatory cytokines and chemokines have been shown to disrupt homeostasis in the cartilage matrix of OA patients [2–4], with increased production of interleukin-1 (IL-1)β and tumor necrosis factor (TNF)α by articular chondrocytes [5] characteristic of established OA. In addition, IL-1β has been shown to induce chondrocytes to produce other inflammatory mediators, including IL-6 and nitric oxide, further amplifying detrimental cellular responses [6]. Furthermore, IL-1β expression results in a downregulation of cartilage extracellular matrix (ECM) components by inhibiting anabolic activities and increasing catabolic activities in chondrocytes, resulting in a pathological degradation of cartilage ECM, the hallmark of OA. The resulting imbalance in matrix metalloproteinase (MMPs) and tissue inhibitor of metalloproteinase (TIMP)-3 is thought to play a critical role in cartilage degradation [7].

In addition to inflammatory cytokines, increased production of reactive oxygen species (ROS) has been seen throughout the joints of OA patients, including the synovium, cartilage, and subchondral bone, further disrupting ECM homeostasis in the articular cartilage. Continuous oxidative stress such as this can lead to cartilage degradation via mitochondria damage, enhanced lipid peroxidation [8–10]. The ability of OA patients to respond to this stress is also compromised, with oxidant scavenging enzymes, such as superoxide dismutase, significantly decreased in OA chondrocytes, as compared to normal chondrocytes [11,12]. Excess production of oxidants is linked with apoptosis of cartilage chondrocytes [13], suggesting the therapeutic potential of antioxidants in OA treatment.

Eupatilin [2-(3,4-dimethoxyphenyl)-5,7-dihydroxy-6-methox- ychromen-4-one] is a pharmacologically active flavone derived from *Artemisia princeps* Pampanini (AP) (family Asteraceae), widely used as an herbal medicine in Asia. Eupatilin was originally developed as a gastroprotective agent for the treatment of gastric mucosal injuries. Interestingly, several *in vitro* and *in vivo* studies have demonstrated anti-inflammatory and oxygen radical scavenging properties of the eupatilin [14–19], suggesting a potential use of this agent beyond its original indication.

Until now, the primary goal of OA therapy has been that of pain relief, treated primarily through the use of nonsteroidal anti-inflammatory drugs (NSAIDs) [20]. Although the therapeutic effects of NSAIDs in OA are well established, chronic use of NSAIDs, including both traditional NSAIDs and selective cyclooxygenase-2 (COX-2) inhibitor, have been associated with increased risk of gastrointestinal (GI) complications, ranging from mild gastritis to serious peptic ulcer bleeding [21,22]. To overcome these complications, some physicians prescribe mucosa-protective agents, including eupatilin, together with NSAID for OA patients complaining of GI symptoms or having high risk of GI side effects.

Given the potential importance of oxidative stress in OA pathogenesis, along with the antioxidative properties of eupatilin, we sought to investigate the effects of eupatilin in an animal model of OA. Specific outcomes associated with eupatilin treatment included pain reduction, the degree of cartilage destruction, and the state of local oxidative damage in a rat model of OA.

## Materials and Methods

### Animals

Six-week-old male Wistar rats weighing 140–230 g at the start of the experiment were purchased from Central Lab Animal Inc. (Seoul, South Korea). The animals were housed three per cage in a room with controlled temperature conditions (21–22°C) and lighting (12 h light/dark cycle) with access to sterile food and water. All animal procedures were approved by the Animal Research Ethics Committee of the Catholic University of Korea (Permit Number: 2013-0015-01).

### Induction of OA in the Rat and Treatment with Eupatilin

Animals were randomly assigned to treatment groups prior to the start of the study. After anesthetization with isoflurane, rats were injected with 3 mg of monosodium iodoacetate (MIA) (Sigma, St. Louis, MO, USA) in a 50 μL volume using a 26.5-G needle inserted through the patellar ligament into the intra-articular space of the right knee; control rats were injected with an equivalent volume of saline. Eupatilin was graciously provided by Dong-A Pharmaceutical Co., Ltd. (Yong-In, Korea) and dissolved in 10% dimethyl sulfoxide (DMSO). Eupatilin was administered orally daily at a dose of 100 mg/kg.

### Assessment of Pain Behavior

MIA-treated rats were randomized to each experimental group. Nociceptive testing was performed using a dynamic plantar esthesiometer (Ugo Basile, Gemonio, Italy), an automated version of the von Frey hair assessment procedure, before MIA injection (Day 0) and on each day thereafter. The rats were placed on a metal mesh surface in an acrylic chamber in a temperature-controlled room (21–22°C) and rested for 15 min before testing. The touch stimulator unit was oriented beneath the animal, and an adjustable angled mirror used to place the stimulating microfilament (0.5 mm diameter) below the plantar surface of the hind paw. When the instrument was activated, a fine plastic monofilament advanced at a constant speed and touched the paw in the proximal metatarsal region. The filament exerted a gradual increasing force on the plantar surface, starting below the threshold of detection and increasing until the stimulus became painful, indicated by the removal of the paw. The force required to elicit a paw withdrawal reflex was recorded automatically, measured in g. A maximum force of 50 g and a ramp speed of 20 s were used for all esthesiometry tests. Pain behavioral tests of secondary tactile allodynia were conducted immediately before administering eupatilin.

### Histological and Immunohistochemical Analyses

Histological changes were assessed to confirm the effect of eupatilin on cartilage degeneration in the knee joints of OA rats. The knee joints, including the patella and joint capsule, were resected and stored in 10% neutral buffered formalin for 48 h at 4°C. The fixed specimens were then decalcified with 5% formic acid for 6 days at 4°C. After decalcification, the specimens were embedded in paraffin. Standardized 7 μM serial sections were obtained at the medial and lateral midcondylar level in the sagittal plane and were stained with hematoxylin and eosin (HE), safranin O-fast green, and toluidine blue to enable evaluation of proteoglycan content. Slides for immunohistochemistry were deparaffinized and rehydrated using a graded ethanol series. The sections were depleted of endogenous peroxidase activity by adding methanolic H_2_O_2_, and then blocked with normal goat serum for 30 min. Samples were incubated overnight at 4°C with antibodies to IL-1β at a dilution of 1:50 (Santa Cruz Biotechnology, Santa Cruz, CA, USA), matrixmetalloproteinase-13 (MMP-13) at 1:50 (Abcam, Cambridge, UK), IL-6 at 1:50 (Abcam), inducible nitric oxide synthase (iNOS) at 1:100 (Abcam), and nitrotyrosine 1:100 (Santa Cruz). The samples were then incubated with their respective secondary antibodies, biotinylated anti-mouse IgG or rabbit IgG, for 20 min, conjugated to a streptavidine peroxidase complex (Vector Laboratories, Burlingame, CA, USA) for 1 h, and finally with 3,30-diaminobenzidine (Dako, Glostrup, Denmark). The sections were counterstained with Mayer’s hematoxylin and photographed using an Olympus photomicroscope (Olympus, Tokyo, Japan).

A modified Mankin’s histological score [23] (original score proposed by Mankin *et al*. [24]) was used to score histological injuries of the articular cartilage as follows. The structure was scored on a scale of 0–6, where 0 = normal; 1 = irregular surface, including fissures into the radial layer; 2 = pannus; 3 = absence of superficial cartilage layers; 4 = slight disorganization (cellular row absent, some small superficial clusters); 5 = fissure into the calcified cartilage layer; and 6 = disorganization (chaotic structure, clusters, and osteoclasts activity). Cellular abnormalities were scored on a scale of 0–3, where 0 = normal; 1 = hypercellularity, including small superficial clusters; 2 = clusters; and 3 = hypocellularity. The matrix staining was scored on a scale of 0–4, where 0 = normal/slight reduction in staining; 1 = staining reduced in the radial layer; 2 = staining reduced in the interterritorial matrix; 3 = staining present only in the pericellular matrix; and 4 = staining absent. Joint space width was estimated measuring the sum of the nearest distance of medial and lateral tibiofemoral joints. Histological evaluation was performed by two independent experienced researchers who were blinded to the treatment arm.

### Microscopic assessment of OA joints

The tibia and femur bones were separated and all excess soft tissue was carefully dissected under a dissecting microscope. The articular surface of each specimen was rinsed with phosphate-buffered saline (PBS). The cartilage surfaces of the femoral condyle and the tibial plateau were examined with a microscope.

### Primary culture and treatment of OA chondrocytes

All relevant protocols were approved by the institutional review board of Bucheon St. Mary’s Hospital (HC14TISI0071), and performed in accordance with the Helsinki II Declaration. Informed, written consent was obtained from all patients. OA was diagnosed using the American College of Rheumatology criteria for this disease [25]. Cartilage samples were washed in calcium- and magnesium-free PBS and finely grounded. Chondrocytes were obtained by digesting the articular cartilage with 0.2% pronase (Sigma) for 1 h, followed by digestion with 0.2% Clostridia collagenase (Sigma) for 3 h at 37°C in high-glucose Dulbecco's modified Eagle medium (DMEM; Life Technologies, Carlsbad, CA, USA) containing an antibiotic–antimycotic solution (100 U/mL penicillin, 100 μg/mL streptomycin, and 0.25 μg/mL amphotericin B; Life Technologies). Undigested cartilage was removed with a 70-μg nylon mesh (Cell Strainer; Falcon), and the chondrocytes were collected by centrifugation. Cells were then washed twice, followed by re-suspension in DMEM supplemented with 10% fetal bovine serum (FBS; Life Technologies). Finally, the cells were plated in 100 mm tissue culture dishes for expansion at 37°C in a 5% CO_2_ humidified atmosphere for 10 days (Shel Lab, Cornelius, OR, USA). Culture medium was changed every 2–3 days. Following expansion, chondrocytes were cultured under FBS-free DMEM conditions (5%, v/v) and were used in monolayers at confluence for all experiments. Cells (1 × 10^5^ cells/well) were placed in 24-well tissue culture plates, and the medium was replaced with serum-free DMEM the following day. Twenty-four hours later, the cells were pretreated with eupatilin for 2 h and then stimulated with or without recombinant human IL-1β (20 ng/mL; R&D Systems) for 48 h.

### MTT(3-(4,5-Dimethylthiazol-2-yl)-2,5-diphenyltetrazolium bromide) assay

Cell viability was assessed using an MTT assay based on the ability of mitochondria of viable cells to convert soluble MTT into an insoluble purple formazan reaction product. Cells were treated with MTT solution (5 mg/mL in Dulbecco’s modified Eagle’s medium (DMEM) without phenol red; Sigma) for 2 h. The MTT solution was then aspirated and replaced with 200 mL/well dimethyl sulfoxide (DMSO). Detection occurred by addition of 100 mL of the reaction mixture, and read at 540 nm. MTT assays for the peripheral blood mononuclear cells were derived from two independent experiments, performed in duplicate.

### Reverse transcription and real-time polymerase chain reaction (PCR)

Total RNA was isolated from human chondrocytes using the TRIzol method (Invitrogen). Complementary DNA (cDNA) was prepared by reverse transcription of single-stranded RNA using the High Capacity cDNA Reverse Transcription Kit (Applied Biosystems), according to the manufacturer's instructions. mRNA expression was estimated using real-time quantitative PCR with LightCycler FastStart DNA Master SYBR green I (Takara), according to the manufacturer's instructions. The primer pairs used in these reactions were as follows: for control human β-actin, forward 5′-GGA CTT CGA GCA AGA GAT GG-3′, reverse 5′-TGT GTT GGC GTA CAG GTC TTT G-3′; for human tissue inhibitor of metalloproteinase (TIMP)-1, forward 5′-AAT TCC GAC CTC GTC ATC AG-3′, reverse 5′-TGC AGT TTT CCA GCA ATG AG-3′; for human ADAMTS5, forward 5′-TAT GAC AAG TGC GGA GTA TG-3′, reverse 5′-TTC AGG GCT AAA TAG GCA GT-3′. The amplification reactions, data acquisition, and analysis were performed with the LightCycler Real-Time PCR system (Roche Diagnostics, Indianapolis, IN, USA), and the relative levels of gene expression were normalized against β-actin.

### Western blotting

Protein was collected from human chondrocytes that were lysed in RIPA buffer containing protease inhibitors. Protein samples were separated by SDS gel electrophoresis and transferred to a nitrocellulose membrane (Amershm Pharmacia Biotech, Buckinghamshire, U. K.). Transferred membranes were stained with primary antibodies to *p*-JNK, JNK and β-actin (all from Santa cruz). The HRP-conjugated secondary antibody was then added.

### Statistical Analysis

Changes in pain behavior were expressed as means ± standard error of the mean (S.E.M.). Histological assessments and pain behaviors were represented using dot plots. One-way analysis of variance followed by Bonferroni’s post-hoc test was used to compare pain and histological scores. The Shapiro-Wilk test and Levene’s test were used to assess the Gaussian distribution and the equality of variance, respectively. Statistical analyses were performed using SPSS statistical software package standard version 16.0 (SPSS Inc., Chicago, IL, USA). P values ≤ 0.05 (two-tailed) were considered significant.

## Results

### Reduced Pain Generation and Cartilage Degradation by Oral Administration of Eupatilin in MIA-induced OA Rats

**Fig 1:**
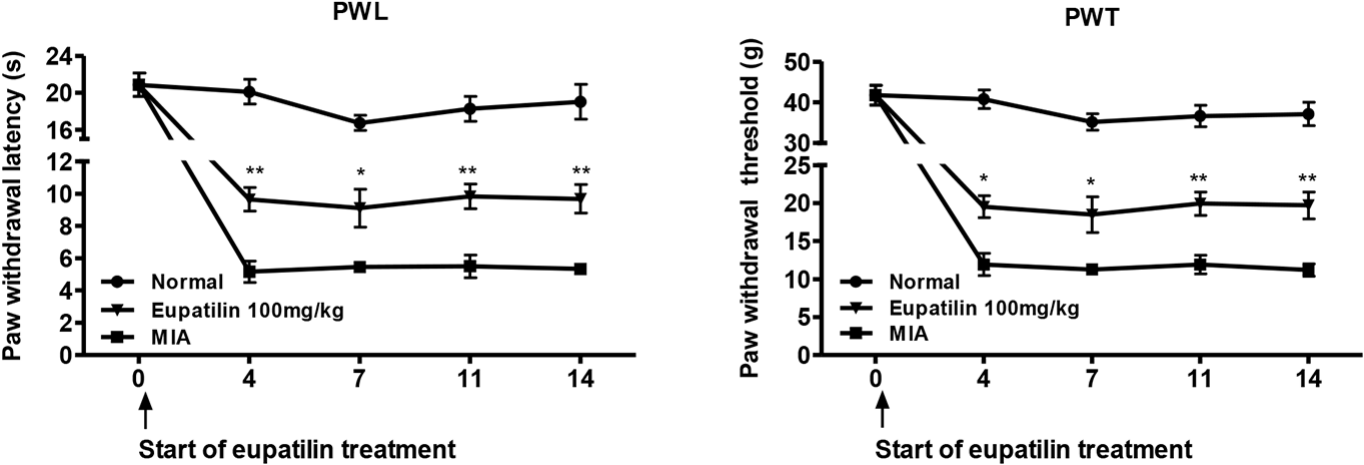
Effects of eupatilin on mechanical hyperalgesia in a model of MIA-induced OA in rats. Rats were injected with 3 mg of monosodium iodoacetate (MIA) in the right knee. Eupatilin (100 mg/kg) was administered orally daily for 14 days after MIA injection. Behavioral tests of mechanical hyperalgesia were evaluated using a dynamic plantar esthesiometer (n = 6 per group per day. Paw withdrawal latency (PWL) and paw withdrawal threshold (PWT) were conducted right before the administration of eupatilin. *P < 0.05, and **P < 0.01 relative to the vehicle-treated MIA-induced OA group.

**Fig 2:**
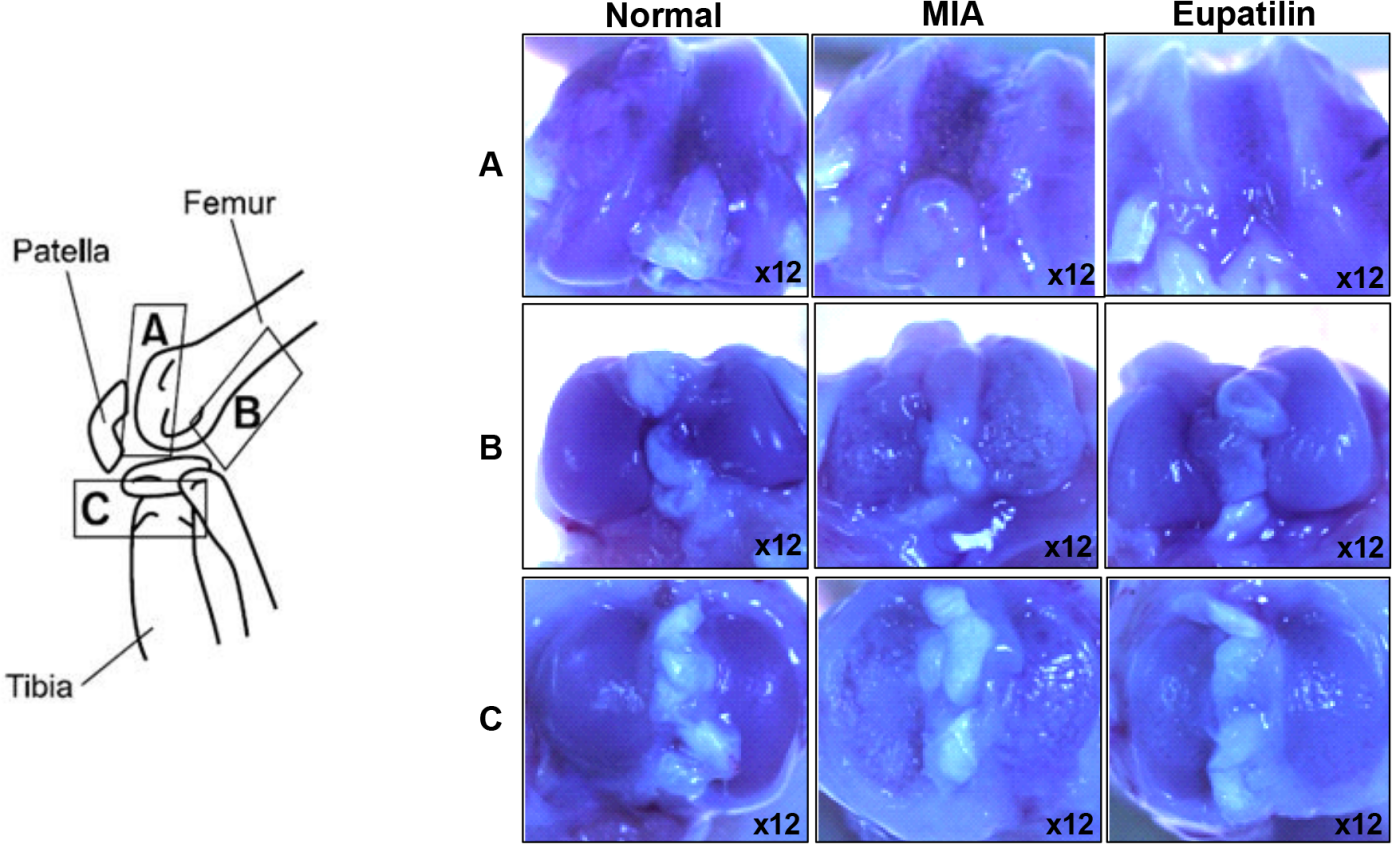
Macroscopic photographs of the damaged articular cartilage after treatment with eupatilin in MIA-induced OA rats. Rats were injected with 3 mg of MIA in the right knee. Eupatilin was administered orally daily for 14 days after MIA injection. The gross morphological changes of the femoral condyles and tibial plateau were photographed using a microscope.

The MIA-induced rat model of OA mimics the pain of human OA, along with the biochemical and structural changes underlying the disease [26]. Because pain is the predominant symptom of OA, we began by evaluating the nociceptive response, followed by a histological examination of the affected tissues. In the von Frey hair assessment test, the paw withdrawal latency (PWL) and the paw withdrawal threshold (PWT) were significantly prolonged in the inflamed hind paw of the rats given oral eupatilin (100 mg/kg), as compared to the placebo group (Fig 1), demonstrating the antinociceptive property of eupatilin in OA rats. On day 7 after intra-articular injection of MIA, oral administration of eupatilin once daily provided an *in vivo* chondroprotective effect that was evident at the macroscopic level. The area of eroded cartilage surface was significantly reduced in the joints of eupatilin-treated rats (Fig 2).

### Chondroprotective Effects of Eupatilin in MIA-induced OA Rats

**Fig 3:**
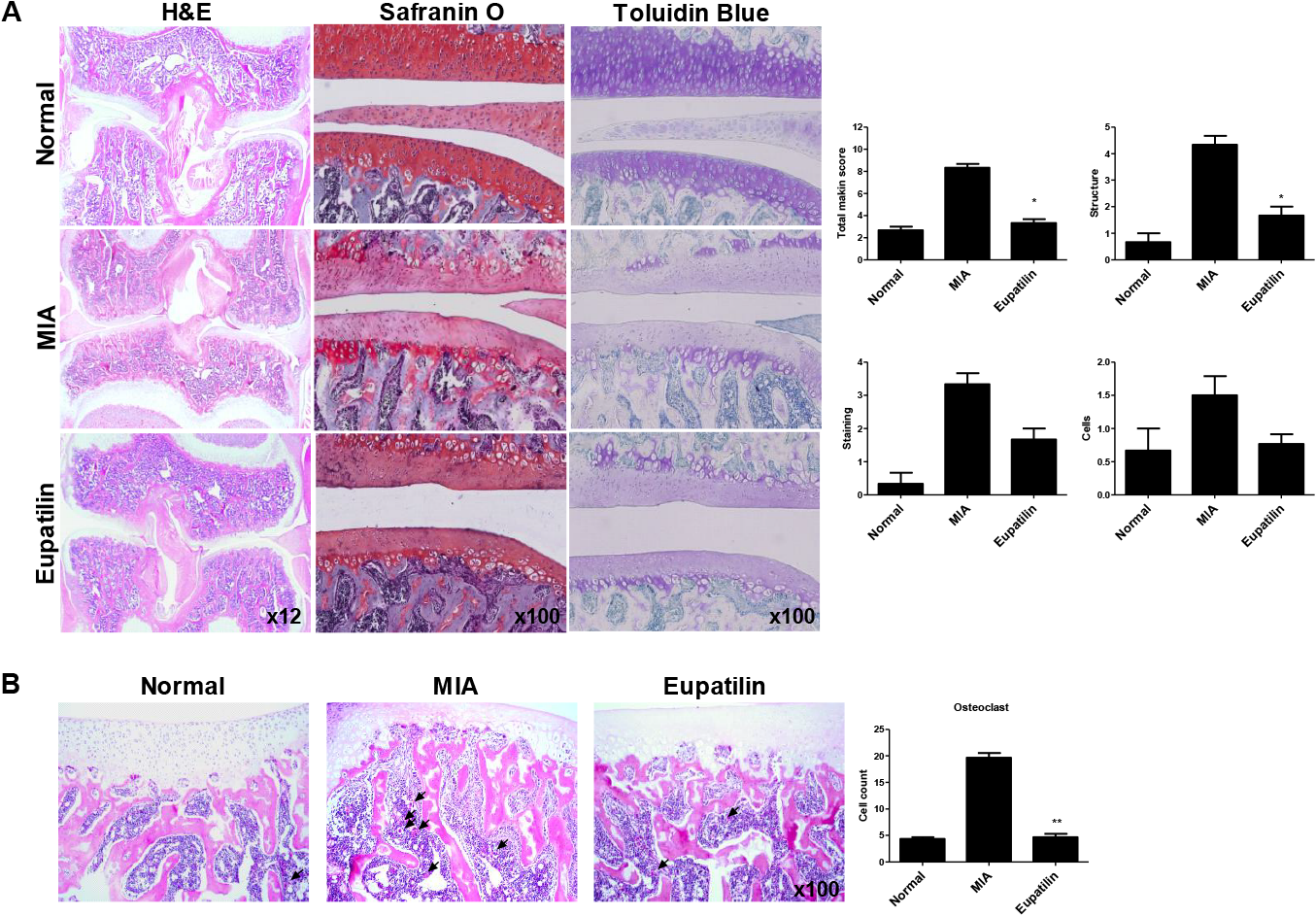
Histological evaluation of joints and osteoclastic activity after treatment with eupatilin in MIA-induced OA. Rats were injected with 3 mg of monosodium iodoacetate (MIA) in the right knee. Eupatilin was administered orally daily for 7 days after MIA injection. (A) The knee joints of OA rats treated with either eupatilin or vehicle control were stained with HE, Safranin O-fast green, and toluidine blue. The joint lesions were graded on a scale of 0–13 using the modified Mankin scoring system, giving a combined score for cartilage structure, cellular abnormalities, and matrix staining. The data are expressed as means ± SEM for six animals per group. (B) Osteoclasts were measured in the subchondral bone region. *P< 0.05, and **P< 0.01 compared with the vehicle-treated MIA-induced OA group.

To evaluate the chondroprotective effect of eupatilin, knee joints from each of the three treatment groups were isolated 7 days after MIA-injection and analyzed microscopically. In the control group, staining revealed smooth articular cartilage and normal cellularity. In contrast, the joints from MIA-induced OA rats showed narrowing in the joint space along with a marked depletion of proteoglycan. These histomorphological changes in the cartilage were significantly reduced in the eupatilin-treated OA animals (Fig 3A). The eupatilin-treated group also showed significantly lower Mankin scores, as compared to vehicle-treated controls (Fig 3A). Involvement of subchondral bone was evident in MIA-induced OA rats, characterized by an increase in multinucleated osteoclasts 7 days after MIA injection [27], which was significantly decreased following eupatilin treatment (Fig 3B).

**Fig 4:**
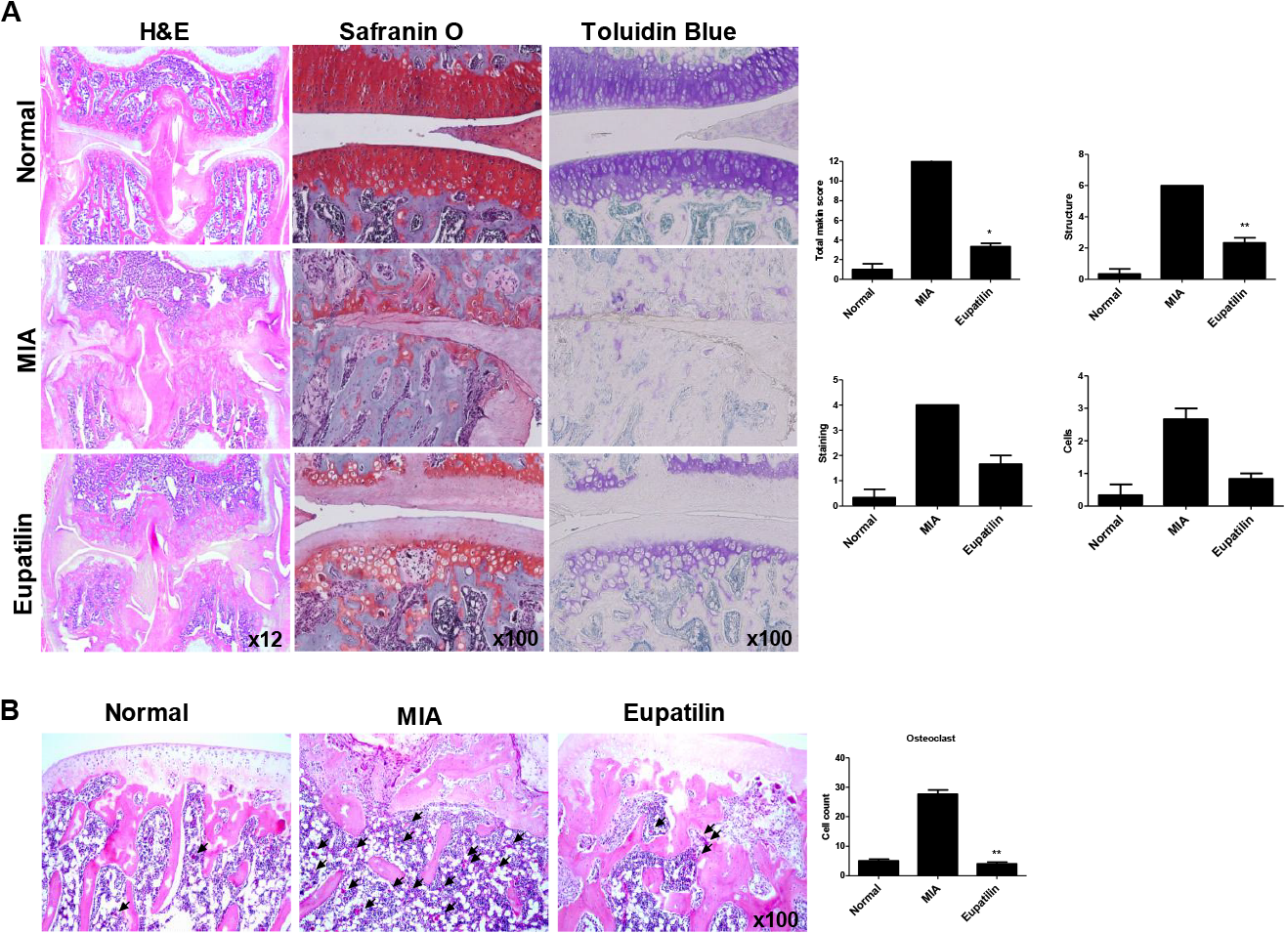
Delayed efficacy of eupatilin in MIA-induced OA. Rats were injected with 3 mg of monosodium iodoacetate (MIA) in the right knee. Eupatilin was administered orally daily for 14 days after MIA injection. (A) The knee joints from the OA rats treated with either eupatilin or vehicle control were stained with HE, Safranin O, and toluidine blue. The joint lesions were graded on a scale of 0–13 using the modified Mankin scoring system, giving a combined score for cartilage structure, cellular abnormalities, and matrix staining. The data are expressed as means ± SEM for six animals per group. (B) Osteoclasts were measured in the subchondral bone region. *P< 0.05, and **P< 0.01 compared with the vehicle-treated OA animals.

Next, to evaluate the chondroprotective effect of eupatilin in OA joints, knee joints were isolated 14 days after MIA-injection. Although the articular cartilage isolated from vehicle-treated OA rats showed marked joint space narrowing and proteoglycan depletion, oral administration of eupatilin protected the articular cartilage from damage, while significantly limiting the number of inflammatory cells (Fig 4A). Furthermore, the rapid proliferation of osteoclasts seen after MIA injection was significantly attenuated following eupatilin treatment (Fig 4B). Taken together, eupatilin appears to exhibit chondroprotective property *in vivo*, with the effects maintained until a comparatively late stage of OA.

### Reduced expression of MMP-13, IL-1β, and IL-6 in the cartilage of eupatilin-treated OA rats

**Fig 5:**
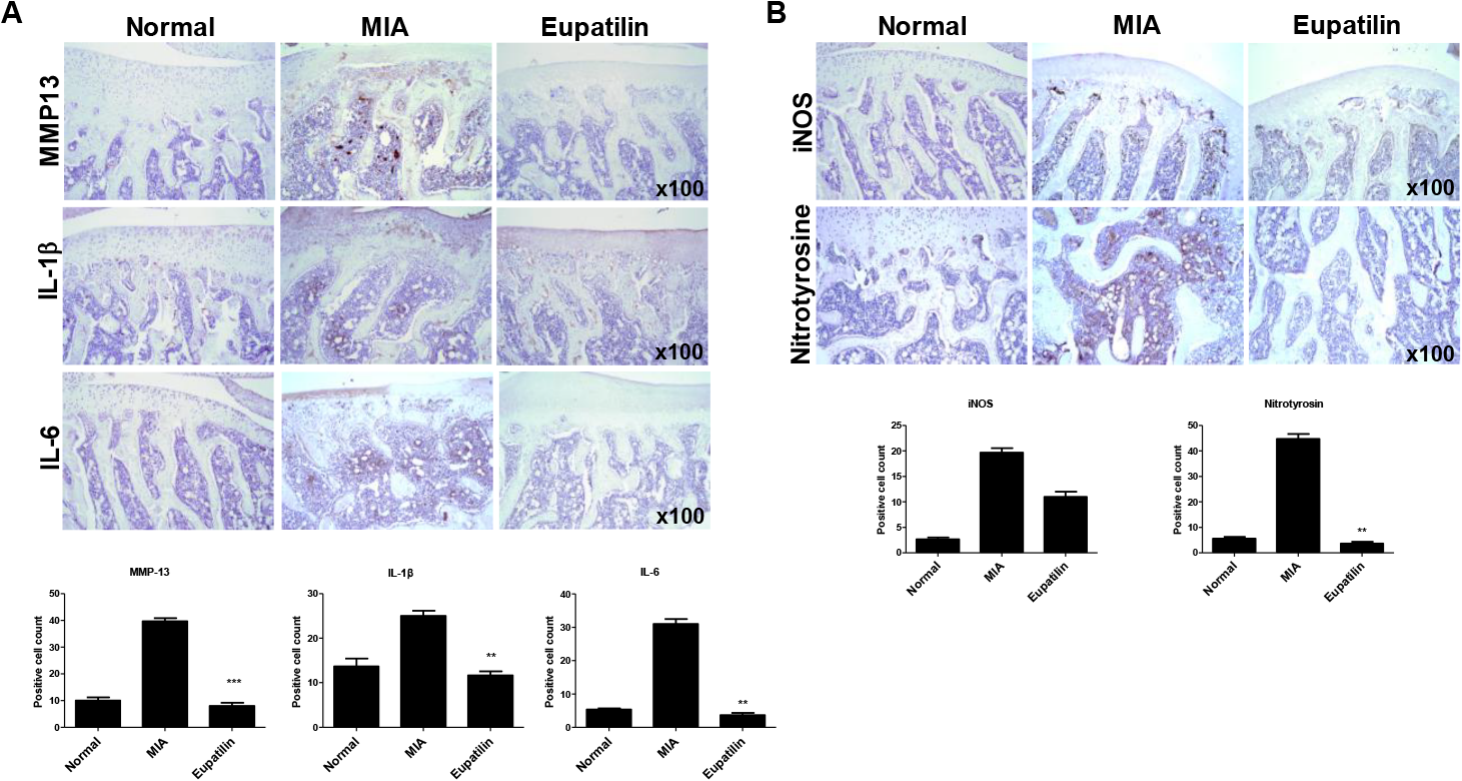
Effects of eupatilin on the expression of MMP-13, IL-1β, IL-6, iNOS, nitrotyrosine in OA joints. Rats were injected with 3 mg of monosodium iodoacetate (MIA) in the right knee. Eupatilin was administered orally daily for 7 days after MIA injection. Immunohistochemical staining was used to identify the expression of MMP13, IL-1β, IL-6 (A), iNOS, and nitrotyrosine (B) in the articular cartilage. The data are expressed as means ± SEM for six animals per group. **P < 0.01 ***P < 0.001 compared with the MIA-induced OA group.

The number of chondrocytes staining positive for MMP-13 was increased in the MIA-induced OA cartilage, with significantly lower percentages of MMP-13-positive chondrocytes evident in the eupatilin-treated group relative to vehicle-treated controls (Fig 5A). In addition to MMP-13, the expression of other proinflammatory cytokines was also examined. Strong induction of IL-1β and IL-6 expression were evident in OA rats; these levels were significantly decreased following eupatilin treatment (Fig 5A).

From a mechanistic standpoint, oxidative stress, through compounds such as nitric oxide (NO), have been shown to mediate enhanced catabolic effects in articular cartilage [28]. NO-associated proteins, such as inducible nitric oxide synthase (iNOS), has been implicated, therefore, in the pathogenesis of OA by contributing to the production of catabolic factors such as IL-1β and MMPs [29]. To determine the degree of oxidative damage in the knee joints of eupatilin-treated OA rats, immunohistochemistry was used to assess the expression of iNOS and nitrotyrosine on day 7 after MIA injection. Strong increases in the expression of iNOS and nitrotyrosine were observed in the articular cartilage of MIA-injected joints, which was abrogated following eupatilin treatment in both the articular cartilage as well as subchondral bone marrow (Fig 5B).

### Effects of eupatilin on the expression of MMP-3, MMP-13, ADAMTS5 and TIMP-1 in human OA chondrocytes

**Fig 6:**
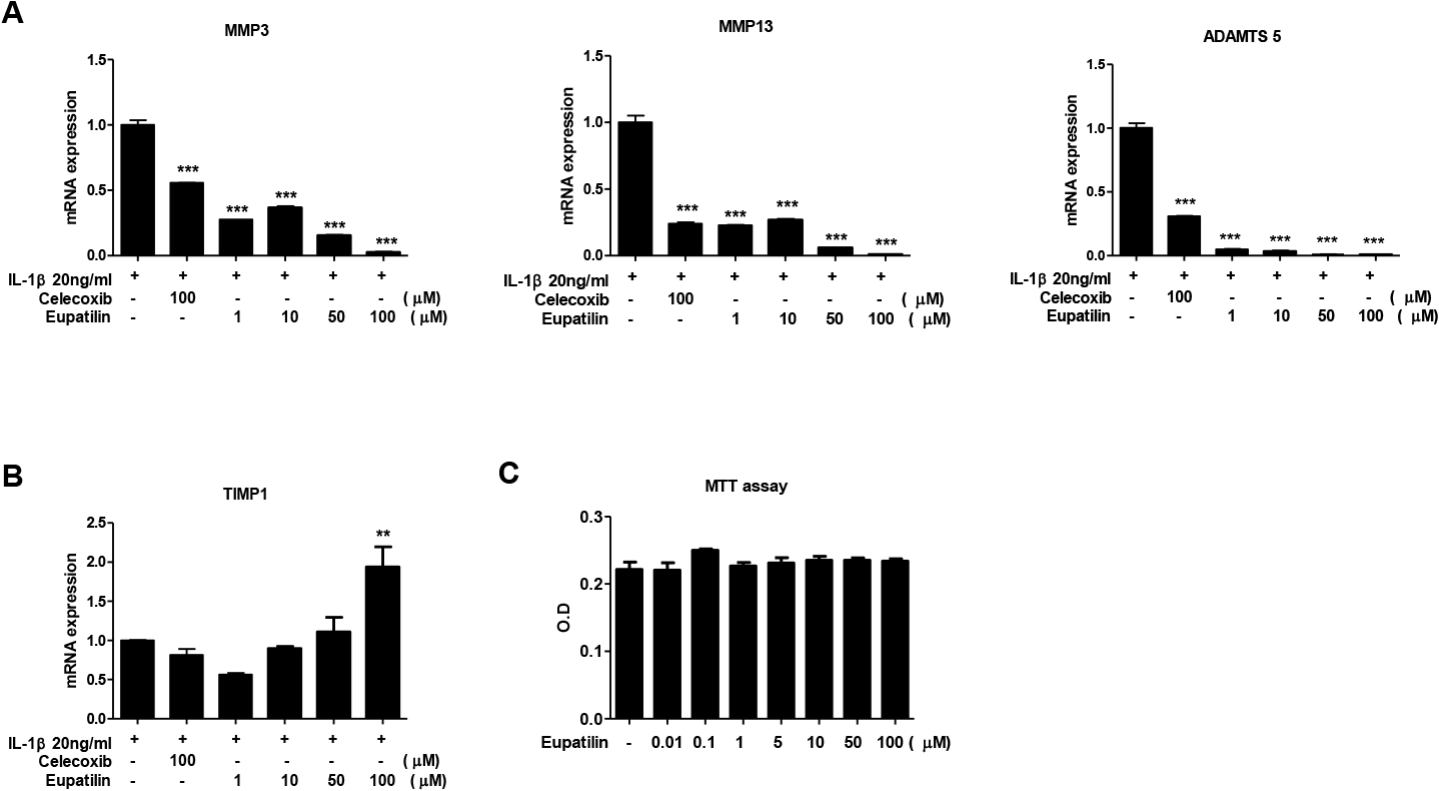
Recovery of anabolic and catabolic activities in human OA chondrocytes following the treatment with eupatilin. Human articular chondrocytes from OA patients were cultured with IL-1β in the presence or absence of eupatilin for 48 h following 24 h in serum-free medium. The mRNA expression of catabolic (MMP-3, and MMP-13, and ADAMTS5) (A) and anabolic (TIMP-1) factor (B) were measured by quantitative real-time PCR; β-actin was used as the internal control. (C) Cell viability was determined by MTT assay. The data are expressed as a mean of three independent experiments per group. **P < 0.01 and ***P < 0.001 compared with IL-1β-stimulated chondrocytes.

We next investigated the effects of eupatilin on the expression of catabolic and anticatabolic molecules in IL-1β-stimulated human OA chondrocytes. Expression of MMP-3, and -13 in IL-1β-stimulated OA chondrocytes were significantly reduced in response to eupatilin treatment in a dose-dependent manner; similar reduction in ADAMTS5 expression in OA chondrocytes were also observed following eupatilin treatment. The inhibitory properties of eupatilin on these catabolic molecules in chondrocytes were comparable with those of celecoxib, a potent inhibitor of MMPs and NO production [30] (Fig 6A). In contrast, expression of anticatabolic factor TIMP-1 in IL-1β-induced OA chondrocytes increased significantly after eupatilin treatment in a dose-dependent manner (Fig 6B). Cell viability, as determined by MTT assay, was not altered at any of the doses used in this study (Fig 6C). Taken together, these data show that eupatilin exert chondroprotective properties *in vivo* by modulating specific catabolic and anticatabolic molecules in OA chondrocytes.

### Effects of eupatilin on JNK activities in human OA chondrocytes

To understand the molecular mechanisms by which effect of eupatilin, we investigated whether the eupatilin modulate JNK signaling pathway in IL-1β-stimulated OA chondrocytes. Phosphorylation of JNK was reduced with eupatilin treatment in a dose dependent manner (data in S1 Fig).

## Discussion

It is becoming increasingly apparent that OA is not simply a degenerative disease, but a multifactorial inflammatory disease involving a host of inflammatory mediators, including IL-1β, IL-6 and NO [31]. Here, we found that oral administration of eupatilin attenuated pain severity, cartilage destruction, joint space narrowing, and reduced oxidative damage in the articular cartilage and the subchondral bone region in an experimentally-induced rat model of OA. These chondroprotective properties were strongly correlated with a reduction in catabolism of the articular cartilage matrix. Specific mediators of cartilage destruction, including multiple MMPs, ADAMTS5, and TIMP1, were all significantly attenuated following eupatilin treatment in both our animal model and in human OA chondrocytes. Similar beneficial effects of eupatilin in OA joints were also ascertained in the delayed phase of OA, suggesting that these findings may be relevant across many stages of the disease.

Although the pathophysiology of OA-related pain has not been fully elucidated, the major goals of OA management are to control pain and to achieve a healthy lifestyle. As the cartilage does not contain nerve endings, it is not a source of OA-related pain. Instead, the generation of joint pain results from the activation of afferent nerve fibers in the subchondral bone, periosteum, synovium, ligament, and joint capsule [32]. The MIA-induced OA murine model is a well-characterized model of painful joint degeneration, in which mechanical hyperalgesia is exhibited [33]. In this OA model, a subchondral bone lesion develops following chondrocytic cell death, consistent with the development of subchondral bone lesions and knee pain in human OA [34–36]. Based upon these similarities, it is reasonable to assume that the attenuated histomorphological changes in subchondral bone lesions in response to eupatilin treatment may contribute to the pain-reducing effects of the drug.

From a mechanistic standpoint, expression of IL-1β, IL-6, iNOS, and nitrotyrosine were all significantly reduced following eupatilin treatment, suggesting that the attenuated cartilage degradation seen in response to the drug was due, in part, to reduced oxidative damage and decreased production of proinflammatory cytokines.

Although the pathophysiology of OA remains poorly understood, inflammatory cytokines, such as IL-1β, IL-6, and IL-17, are all strongly associated with the disruption of homeostasis of ECM components seen in OA pathogenesis [6]. MMPs are capable of degrading all components of the ECM, and their enhanced activity has been strongly implicated in the cartilage degeneration seen in OA joints. Proinflammatory cytokines, such as IL-1β, upregulate MMP expression in articular chondrocytes, which contribute to cartilage destruction [37–39]. Furthermore, IL-1β is able to break down both type 2 collagen, the primary form of collagen found in articular cartilage, and aggrecan. In contrast, TIMPs are endogenous regulators of MMP activity and may represent an anticatabolic strategy in OA maintenance. Our results showed that the expression of MMP-3, and -13, and ADAMTS5 in IL-1β-stimulated human OA chondrocytes decreased significantly after the treatment with eupatilin, whereas TIMP-1 increased. Taken together, these results suggested that eupatilin constitutes a promising option for the treatment of OA.

Continuous, low-grade oxidative stress is one of the primary mechanisms underlying OA pathogenesis [40]. The increase in oxidative stress associated with aging has been shown to predispose human chondrocytes to oxidant-mediated cell death through the dysregulation of antioxidant system [41]. Other mechanisms of oxidative stress include increased production of NO, which can lead to apoptotic insults to human chondrocytes via mitochondrial dysfunction [42]. Physical exercise is able to protect against some of this damage by reducing oxidative stress in the articular capsule, leading to preservation of proteoglycan contents in the articular cartilage in MIA-induced OA rats [43]. Consistent with this observation, Jiang *et al*. recently found that the underlying mechanisms of chondrocyte apoptosis in MIA-induced OA rats are primarily via ROS production and mitochondria-mediated caspase-3 activation [44]. Taken together, these data strongly implicate ROS as a potential target in treating patients with OA.

Following the recent studies, eupatilin inhibits MAPK, NF-κB signaling pathway [16,45]. Our study demonstrated to effect of eupatilin for JNK phosphorylation in IL-1β stimulated chondrocytes and Phosphorylation of JNK was reduced with eupatilin treatment in a dose dependent manner. Further molecular studies, such as determination of whether each interaction requires unique factors, are needed to understand.

In conclusion, eupatilin treatment attenuated the severity of articular cartilage degradation in an MIA-induced OA model, in part through a reduction in oxidative damage, and by shifting the balance of ECM homeostasis toward anabolism both *in vivo* and *in vitro*. With its combination of antioxidative and anti-inflammatory properties, eupatilin may constitute a promising therapeutic option for the management of OA.

## Supporting Information

S1 Fig Effect of eupatillin on the phosphorylation of JNK in human OA chondrocytes. Protein from OA patients of articular chondrocytes were pretreated in absence or presence of eupatilin for 1h with starvation then stimulated with IL-1β for 20min. Expression levels of *p*-JNK, JNK, β-actin was determined by Western blotting. The data are expressed with mean (bar) for three independent experiments. ***P < 0.001 compared with IL-1β-stimulated chondrocytes.
(PDF)
