# Supplementary figures and images for: Eupatilin Exerts Antinociceptive and Chondroprotective Properties in a Rat Model of Osteoarthritis by Downregulating Oxidative Damage and Catabolic Activity in Chondrocytes
Source: PLoS One. 2015 Jun 17;10(6):e0130882. doi: 10.1371/journal.pone.0130882 (PMC4471346; doi:10.1371/journal.pone.0130882)

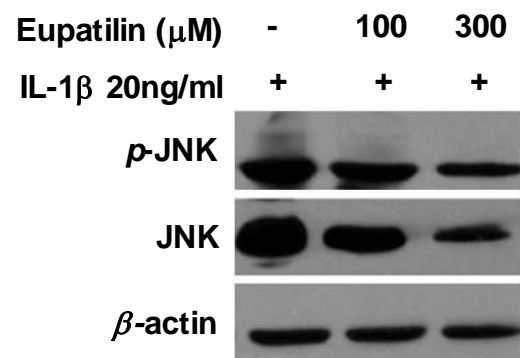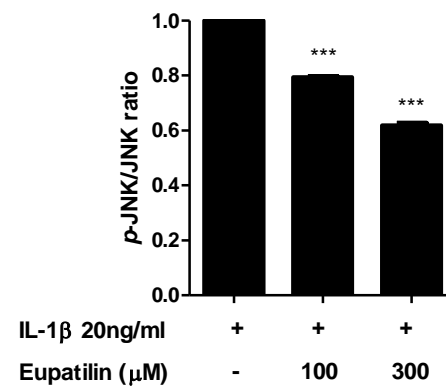

Supplement: S1 Fig — Protein from OA patients of articular chondrocytes were pretreated in absence or presence of eupatilin for 1h with starvation then stimulated with IL-1β for 20min. Expression levels of p-JNK, JNK, β-actin was determined by Western blotting. The data are expressed with mean (bar) for three independent experiments. ***P < 0.001 compared with IL-1β-stimulated chondrocytes. (PDF) [file pone.0130882.s001.pdf]
